# Commentary: Arginine Vasotocin Preprohormone Is Expressed in Surprising Regions of the Teleost Forebrain

**DOI:** 10.3389/fendo.2018.00063

**Published:** 2018-03-01

**Authors:** Jasmine L. Loveland, Caroline K. Hu

**Affiliations:** ^1^Behavioural Genetics and Evolutionary Ecology Research Group, Max Planck Institute for Ornithology, Seewiesen, Germany; ^2^Department of Organismic and Evolutionary Biology, Howard Hughes Medical Institute, Harvard University, Cambridge, MA, United States; ^3^Department of Molecular and Cellular Biology, Howard Hughes Medical Institute, Harvard University, Cambridge, MA, United States

**Keywords:** arginine vasotocin, arginine vasopressin, nonapeptides, preoptic area, hypothalamus, teleost, amygdala

Recently, Rodriguez-Santiago et al. (1) reported that arginine vasotocin (AVT) preprohormone mRNA is expressed in pallial and subpallial nuclei of the cichlid fish, *Astatotilapia burtoni*. Specifically, they claim expression in putative homologs of the mammalian extended amygdala, hippocampus, striatum, and septum. The authors present these results as “surprising” because, to date, this expression has never been reported even when the same methods [i.e., *in situ* hybridization (ISH)] were applied to *A. burtoni* (2) and other fish species [see Table 1 in Ref. (1) in addition to zebrafish (3) and catfish (4)]. We argue that making claims that contradict longstanding conclusions in the field of teleost vasotocin-associated neuroanatomy and neurochemistry warrants better evidence. We identify technical issues regarding *in situ* specificity, qPCR design, and reporting. Importantly, we also point out statements where the authors miscite previous research regarding brain region homologies of proposed vasotocin and vasopressin (AVP) cell populations.

## *In Situ* Hybridization

The authors’ first line of evidence for AVT preprohormone expression in areas of the telencephalon is from non-radioactive ISH of the 3’ UTR of the AVT transcript (Experiment 1). They speculate that previous researchers failed to detect pro-AVT expression outside of the preoptic area (POA) and hypothalamus because of (i) early termination of exposure times to avoid overdevelopment of signal in POA populations and (ii) not examining beyond these focal regions. We find the *lack* of signal contrast between their POA and non-POA populations to be incongruous with having very high and very low expression, respectively. Besides antisense and sense probe treatments, additional evidence is warranted to support the specificity of their abundant signal. First, while no single control on wild-type tissue can definitively demonstrate specificity, the following approaches would all lend needed support: hybridization with a second pro-AVT probe to demonstrate signal overlap, and double labeling of pro-AVT mRNA and either AVT or neurophysin peptides (5) to show co-localization. However, a lack of AVT-immunoreactivity in non-POA areas of the telencephalon would still not be sufficient to prove that pro-AVT mRNA *in situ* staining is artifactual. A third control could be hybridization for mRNAs of known restricted expression (e.g., *GnRH1*) to show their protocol is sound, and that even long color development times do not produce staining artifacts that could be interpreted as false positives, though probes for different genes may not have the same types of background artifact as their pro-AVT probe.

Second, non-radioactive ISH for pro-AVT mRNA outside of the POA has been performed in at least two other studies (3, 4), neither of which the authors cite because they claimed only radioactive ISH evidence existed. In both studies, brain pro-AVT mRNA was found to be restricted to the POA and hypothalamus in zebrafish (3) and catfish (4). Furthermore, a detailed report of AVT expression in an AVT-EGFP transgenic medaka line also shows no AVT-expressing cell bodies in the telencephalon (6). Thus, we find both the ISH data presented and its interpretation, as well as the omission of several key studies (3, 4, 6–10) to be problematic.

## Quantitative PCR

The authors’ second line of evidence is qPCR from a microdissected pallial nucleus that previously has not been shown to express pro-AVT mRNA (Experiment 2). They describe this expression as “lowly abundant,” however, it is difficult to evaluate the specificity and relative abundance of this signal. It would have been exceedingly useful to provide a positive (e.g., POA) and negative (e.g., brain region without pro-AVT ISH signal) control for their qPCR. It would also have been informative to present this data as “relative expression” similar to their previous use of these primers on POA tissue (11). Furthermore, extended cycling in PCR risks amplification of contaminants and artifacts. Their primers flanked exon–exon boundaries; however, the intron between the two target exons is only 74 bp long. Therefore, a 172 bp amplicon could potentially be generated from genomic DNA or pre-mRNA despite a relatively short extension time (e.g., 30 s). These concerns could be alleviated if the authors had adhered to the Minimum Information for Publication of Quantitative Real-Time PCR Experiments (MIQE) Guidelines and reported, as is commonly done, amplicon sequencing and melt curves, correlation coefficients of standard curves, gene efficiencies, and no template and/or no reverse transcriptase controls (12).

## Brain Region Homologies

We have two serious concerns regarding the authors’ representation and interpretation of the fish AVT literature. First, the authors state that AVT neurons have been localized in the POA and anterior tuberal nucleus (aTn) and cited (2, 13) as references. However, the non-POA AVT neurons that these two cited papers report are *not* in the anterior tuberal nucleus. In most reports, in other fish species, these cells are localized in the ventral tuberal nucleus, although the nomenclature used in Ref. (2) was lateral tuberal nucleus and in Ref. (13) was ventral tuberal hypothalamus. The distinction between anterior and ventral tuberal nuclei is well established and non-trivial, because the aTn is a partial homolog to the mammalian ventromedial hypothalamus, whereas the ventral tuberal nucleus is a partial homolog to the mammalian anterior hypothalamus (13–17). Second, Rodriguez-Santiago et al. (1) stated “the magnocellular and gigantocellular AVT neuron populations are hypothesized to be homologous to the supraoptic nucleus in tetrapods […] while the parvocellular cell group is the putative homolog of the PVN of the mammalian POA” (pg. 4) and cited in support (16, 18–20). There are several problems with this statement. Both Kapsimali et al. (18) and Olivereau et al. (19) actually proposed that only magnocellular AVT cells are homologous to those in the *mammalian PVN, not those in the supraoptic nucleus*. In fact, Kapsimali et al. (18) concluded that patterns of Nurr1 expression support the hypothesis that the preoptic magnocellular cells in medaka are homologous to cells in the mammalian paraventricular nucleus (PVN). Notably, Moore and Lowry (20), the only cited source that supports the view of putative homology stated by Rodriguez-Santiago et al. (1), prefaced their proposed homologies with “[these homologies] should be viewed as proposing tentative hypotheses of homology that need to be investigated more thoroughly” (pg. 252). Altogether, it seems as though Rodriguez-Santiago et al. (1) adopted the proposed AVT/AVP homologies by Moore and Lowry (20), but made the mistake of justifying it with papers that *do not actually support this view*. Altogether, the authors’ misnaming of hypothalamic (tuberal) nuclei and incorrectly citing previous research misleads readers, especially those who may not be familiar with teleost neuroanatomy.

In conclusion, while we agree with the authors that research on vasotocin function and evolution benefits from comparative studies of nonapeptide systems—particularly the distributions of vasotocin receptors and vasotocin projections—caution should be exercised to ensure that the data are robust enough to support the claims.

## Author Contributions

JLL conceived of the commentary. JLL and CKH researched the manuscript. JLL drafted the manuscript. JLL and CKH revised and edited the manuscript.

## Conflict of Interest Statement

The authors declare that the research was conducted in the absence of any commercial or financial relationships that could be construed as a potential conflict of interest.
