# Corrigendum: Commentary: Arginine Vasotocin Preprohormone Is Expressed in Surprising Regions of the Teleost Forebrain

**DOI:** 10.3389/fendo.2018.00157

**Published:** 2018-04-06

**Authors:** Jasmine L. Loveland, Caroline K. Hu

**Affiliations:** ^1^Behavioural Genetics and Evolutionary Ecology Research Group, Max Planck Institute for Ornithology, Seewiesen, Germany; ^2^Department of Organismic and Evolutionary Biology, Howard Hughes Medical Institute, Harvard University, Cambridge, MA, United States; ^3^Department of Molecular and Cellular Biology, Howard Hughes Medical Institute, Harvard University, Cambridge, MA, United States

**Keywords:** arginine vasotocin, arginine vasopressin, nonapeptides, preoptic area, hypothalamus, teleost, amygdala

In the original Commentary article on page 1, the last name of a co-author of the article that was commented on was misspelled as “Winston” instead of “Winton.” The authors apologize for the mistake. This error does not change the scientific conclusions of the Commentary in any way.

The original article has been updated.

## Conflict of Interest Statement

The authors declare that the research was conducted in the absence of any commercial or financial relationships that could be construed as a potential conflict of interest.

